# Clinical implications of choroidal vascular brightness using ultra-widefield indocyanine green angiography in polypoidal choroidal vasculopathy

**DOI:** 10.1038/s41598-023-31745-y

**Published:** 2023-04-19

**Authors:** Areum Jeong, Xue Yao, Kyungmin Lee, Sang Hyun Park, Min Sagong

**Affiliations:** 1 Department of Ophthalmology, Yeungnam University College of Medicine, #170 Hyunchungro, Nam-Gu, Daegu, 42415 South Korea; 2 Yeungnam Eye Center, Yeungnam University Hospital, Daegu, South Korea; 3 Department of Robotic Engineering, DGIST, #333, Techno Jungang-Daero, Dalseong-Gun, Daegu, South Korea

**Keywords:** Retina, Macular degeneration

## Abstract

Polypoidal choroidal vasculopathy (PCV) is characterized by choroidal vascular abnormalities including polypoidal lesion and branching vascular networks. Not only choroidal structural changes, but also choroidal hyperpermeability and congestion are also thought to be involved in pathogenesis of PCV. We investigated choroidal vascular brightness intensity (CVB) using ultra-widefield indocyanine green angiography (UWF-ICGA) images and analyzed its association with clinical features in patients with PCV. In this study, 33 eyes with PCV and 27 eyes of age-matched controls were included. CVB was measured by extracting the enhanced pixels of choroidal vessels after the reference brightness across the images was adjusted to be uniform. Associations between choroidal vascular features and the clinical features of PCV were also determined. The mean CVB was higher in PCV than control eyes, regardless of the segmented region (all p < 0.001). CVB was also higher at the posterior pole than at the periphery, and the inferior quadrants were brighter than the superior quadrants in both the PCV and control group (all p < 0.05). In affected eyes, CVB was higher than in unaffected fellow eyes at the posterior pole, whereas there was no difference at the periphery. Posterior pole CVB correlated significantly with subfoveal choroidal thickness (r = 0.502, p = 0.005), polyp number (r = 0.366 p = 0.030), and the greatest linear dimension (r = 0.680, p = 0.040). Greatest linear dimension was positively correlated with CVB at posterior pole (p = 0.040), whereas SFCT or CVD in all regions didn't show the significant correlation. The UWF ICGA results showed an increase in CVB at the inferior quadrants and posterior pole, suggesting venous outflow congestion in PCV eyes. CVB might provide more substantial information on the phenotype than other choroidal vascular features.

## Introduction

Pachychoroid disease spectrum is defined as a group of clinical entities characterized by similar choroidal findings, clinical features, and pathogenesis^1^. Choroidal congestion and choroidal vascular hyperpermeability, as well as attenuation of the choriocapillaris, are common structural and functional abnormalities in a range of clinical entities developing from the same pathological process^2^. While choroidal imaging has been increasingly used since the introduction of the concept of pachychoroid disease spectrum, many questions regarding their pathogenesis and evolution, and the mechanism of disease progression, remain unanswered.

Several studies based on optical coherence tomography (OCT) and conventional indocyanine green angiography (ICGA) have demonstrated the associations of choroidal hyperpermeability and increased subfoveal choroidal thickness (SFCT) with choroidal venous dilation^2,3^. These results suggest that choroidal congestion contributes to the pathogenesis of polypoidal choroidal vasculopathy (PCV). In addition, a recent study that evaluated structural OCT scans of the choroid of the macular region reported the presence of vortex vein anastomosis^4^. However, en face OCT scans have focused on the posterior pole of the fundus, which does not represent the entire vortex vein drainage system; images acquired in this area only allow analysis of the terminal branches of the vortex veins.

Recent advances in multimodal imaging and ultra-widefield (UWF)-ICGA have enabled visualization of the entire fundus, allowing for more detailed assessment of the morphological features and extent, as well as quantification of the vortex vein. Spaide et al. evaluated choroidal vascular patterns from the entire fundus using UWF-ICGA and observed intervortex venous anastomosis, which implies increased venous pressure with outflow impediment from the choroid together with abnormalities in the vortex vein^5^. Jeong et al. reported macular extension of an engorged vortex vein as a common finding in PCV, and a significant association thereof with a larger choroidal hyperpermeability area and greater thickness of Haller's layer. These observations indicate that venous outflow disturbance and overloading may lead to PCV^6^. Several parameters such as choroidal vascular density and choroidal vascular index have been proposed and found to be correlated with choroidal hyperpermeability or treatment response^7,8^. However, those are qualitative and only reflect a static structure rather than dynamic changes in blood flow and does not accurately describe the hemodynamic characteristics of the entire choroid.

In this study, we investigated the choroidal vascular brightness intensity (CVB) as a new parameter for quantitative evaluation of the degree of choroidal congestion. We also analyzed the association of choroidal vascular features including CVB with the clinical phenotypes of PCV.

## Results

### Baseline characteristics

The study included 33 eyes of 31 PCV patients and 27 eyes of 27 age-matched normal controls. There were no significant differences in age, sex, or spherical equivalent between the two groups. The mean BCVA was 0.83 ± 0.50 logMAR in the PCV group and 0.05 ± 0.06 logMAR in the control group; the difference was significant (p < 0.001). In the PCV group, the mean greatest linear dimension was 2,657.2 ± 906.3 μm and the mean number of polyps was 2.76 ± 0.82 (Table 1).

**Table 1:** Baseline characteristics of the PCV patients and normal controls.

Variables	PCV (n = 33 eyes)	Control (n = 27 eyes)	*p*-value
Age (years)	68.4 ± 9.1	66.3 ± 10.2	0.168*
Male/Female	18 (54.5%)/15 (45.5%)	15 (55.6%)/12 (44.4%)	0.539^†^
Spherical equivalent (diopter)	− 0.23 ± 0.98	− 0.27 ± 0.86	0.401*
Baseline BCVA (logMAR)	0.826 ± 0.497		
SFCT (μm)	365.67 ± 77.95	276.42 ± 59.87	< 0.001*
Haller’s layer	279.22 ± 59.62	180.65 ± 51.68	< 0.001*
Choriocapillaris/Sattler’s layer	86.44 ± 21.43	94.42 ± 52.19	0.022*
Central macular thickness (μm)	392.4 ± 114.8	229 ± 30.86	0.001*
Greatest linear dimension (μm)	2657.2 ± 906.3		
Polyp number	2.76 ± 0.82		

*BCVA* best-corrected visual acuity, *logMAR* logarithm of the minimum angle of resolution, *PCV* polypoidal choroidal vasculopathy, *SFCT* subfoveal choroidal thickness.

*Mann–Whitney U-test.

^†^Fisher’s exact test.

### Comparison of choroidal vascular features between the PCV and normal control groups

The mean SFCT was significantly thicker in the PCV than control group (365.67 ± 77.95 vs. 276.42 ± 59.87 μm; p < 0.001), as was the Haller layer (279.22 ± 59.62 vs. 180.65 ± 51.68 μm; p < 0.001). By contrast, the choriocapillaris/Sattler layer was thinner in the PCV than control group (86.44 ± 21.43 vs. 94.42 ± 52.19 μm; p = 0.022) (Table 1). The mean CVB was higher in PCV than control eyes regardless of the segmented region (all p < 0.001). Additionally, CVB was higher at the posterior pole than periphery, whereas the inferior quadrants were brighter than the superior quadrants in both the PCV and control group (all p < 0.05). The mean CVD was higher in PCV than controls (p = 0.002). Higher CVD in patients with PCV was also found in posterior pole and peripheral region (p = 0.030 and p = 0.010) (Table 2).

**Table 2:** Regional distribution of choroidal vascular features in patients with PCV and normal controls.

	PCV (n = 33 eyes)	Control (n = 27 eyes)	*p*-value
Choroidal vascular brightness intensity			
Total	105.34 ± 21.68	89.80 ± 22.54	< 0.001
Posterior pole	112.82 ± 28.97	103.31 ± 29.80	< 0.001
Peripheral region	102.75 ± 20.28	96.13 ± 23.37	< 0.001
Superior	94.37 ± 23.10	89.34 ± 21.65	< 0.001
Superotemporal	85.34 ± 22.78	82.77 ± 19.91	< 0.001
Superonasal	83.71 ± 20.19	81.34 ± 28.68	< 0.001
Inferior	111.51 ± 25.65	103.25 ± 24.38	< 0.001
Inferotemporal	104.13 ± 22.14	99.67 ± 21.98	< 0.001
Inferonasal	101.34 ± 24.09	95.40 ± 27.72	< 0.001
Choroidal vascular density			
Total	26.37 ± 2.12	24.68 ± 2.49	0.002
Posterior pole	25.85 ± 2.09	23.76 ± 1.97	0.030
Peripheral region	28.59 ± 2.65	26.80 ± 2.83	0.010

*PCV* polypoidal choroidal vasculopathy.

### Comparison of choroidal vascular features between affected and unaffected fellow eyes in unilateral PCV

**Figure 1:**
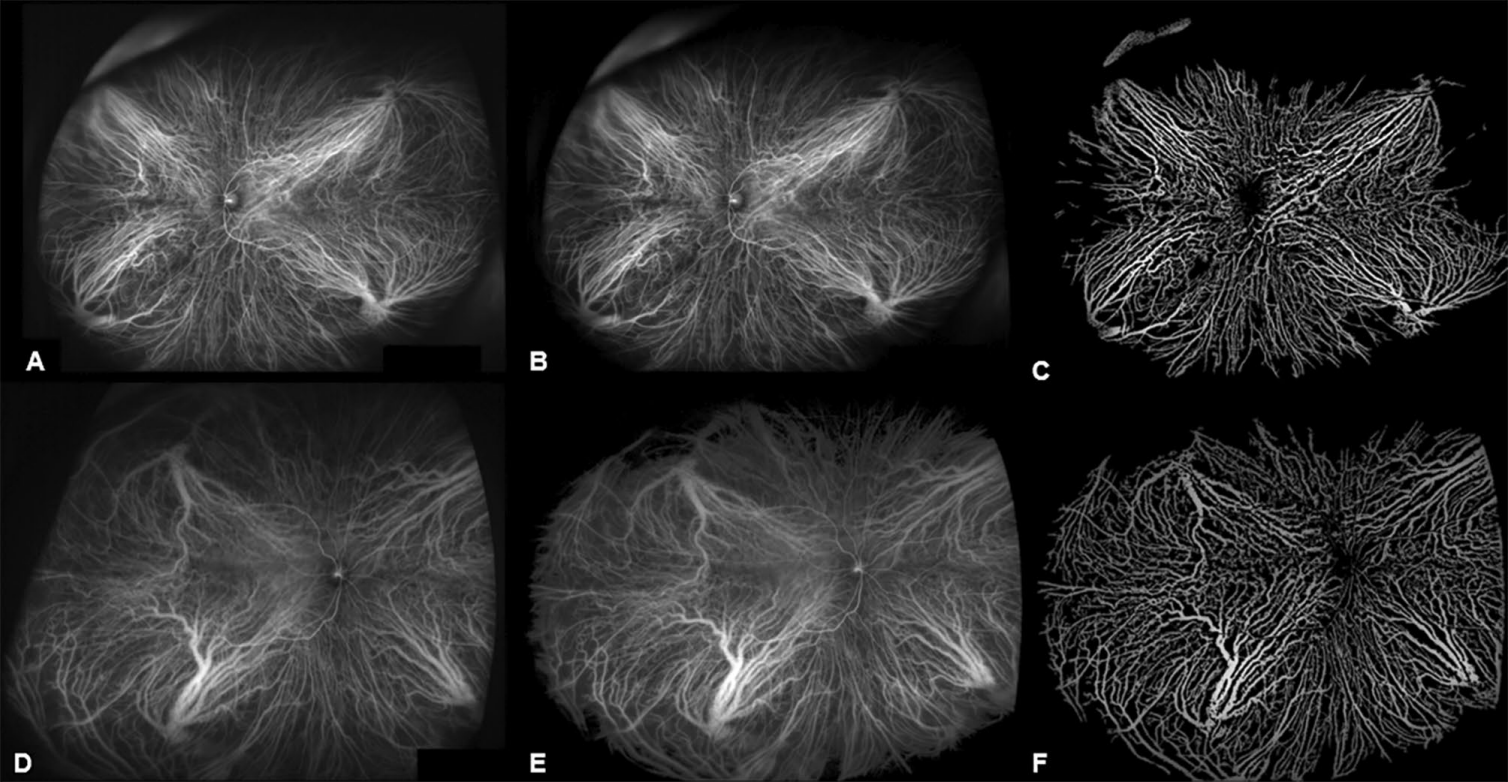
Comparative analysis of choroidal vascular brightness intensity (CVB) between affected left eye and unaffected right eye in a 66-year-old male patient with unilateral PCV. The original ICGA image of affected left eye was cropped to remove the areas obscured by the eyelashes (**A**). The images were adjusted based on the brightness of the retinal blood vessels at the optic nerve location to compensate for the difference in brightness between the images of each patient (**B**). Image thresholding method was applied to extract enhanced pixels. This method was used to extract the brightness of only the choroidal vessels. The mean CVB of entire fundus converted to 8-bit gray scale was 112.56. The CVB of affected left eye in the posterior pole and peripheral region were 118.15 and 95.08 respectively (**C**). The same image processing was applied to the unaffected right eye of the same patient (**D**–**F**). The mean CVB of entire fundus in unaffected right eye was 102.75. The CVB of unaffected right eye in the posterior pole and peripheral region were 105.39 and 94.96 respectively.

The affected and unaffected fellow eyes from the 29 patients with unilateral PCV were compared. The results showed that the SFCT was thicker in affected than unaffected fellow eyes (367.94 ± 75.56 vs. 339.95 ± 64.78 μm; p = 0.001), especially in the Haller layer (281.54 ± 57.61 vs. 254.68 ± 50.92 μm; p = 0.002). In the choriocapillaris/Sattler layer, the two eyes did not differ significantly (86.44 ± 21.98 vs. 85.72 ± 22.74 μm; p = 0.148). Affected eyes had a significantly higher mean CVB of the entire fundus than unaffected fellow eyes (110.88 ± 20.81 vs. 101.69 ± 22.75; p = 0.036). There was no difference in CVB in the peripheral region between affected and unaffected eyes (94.19 ± 22.73 vs. 93.98 ± 21.80; p = 0.274), whereas CVB at the posterior pole was significantly higher in affected than unaffected fellow eyes (116.69 ± 28.98 vs. 104.15 ± 23.35; p = 0.001). CVD of affected eyes did not differ significantly from that of unaffected fellow eyes (Table 3). Representative images showing the process of analyzing CVB in unilateral PCV patient are shown in Fig. 1.

**Table 3:** Comparison of choroidal parameters between affected and unaffected fellow eyes in unilateral PCV.

Variables	Unilateral PCV (n = 29 eyes)		
	Affected eye	Unaffected fellow eye	*p*-value
Subfoveal choroidal thickness (μm)	367.94 ± 75.56	339.95 ± 64.78	0.001
Haller’s layer	281.54 ± 57.61	254.68 ± 50.92	0.002
Choriocapillaris/Sattler’s layer	86.44 ± 21.98	85.72 ± 22.74	0.148
Choroidal vascular brightness intensity			
Total	110.88 ± 20.81	101.69 ± 22.75	0.036
Posterior pole	116.69 ± 28.98	104.15 ± 23.35	0.001
Peripheral region	94.19 ± 22.73	93.98 ± 21.80	0.274
Choroidal vascular density			
Total	26.28 ± 2.65	26.10 ± 2.34	0.316
Posterior pole	25.76 ± 2.11	25.97 ± 2.60	0.265
Peripheral region	29.05 ± 2.74	28.98 ± 2.26	0.615

PCV polypoidal choroidal vasculopathy.

### Comparison of choroidal vascular features between unaffected fellow eyes of unilateral PCV and normal controls

The SFCT was thicker in unaffected fellow eyes than normal control group (339.95 ± 64.78 vs. 276.42 ± 59.87 μm; p = 0.001), especially in the Haller layer (254.68 ± 50.92 vs. 180.65 ± 51.68 μm; p = 0.010). The thickness of choriocapillaris/Sattler layer was thinner in unaffected fellow eyes than normal control group (85.72 ± 22.74 vs. 94.42 ± 52.19 μm; p = 0.042). Unaffected fellow eyes had a significantly higher mean CVB of the entire fundus than normal control group (101.69 ± 22.75 vs. 89.80 ± 22.54; p = 0.012). There was no difference in CVB in the peripheral region between unaffected fellow eyes and normal control group (93.98 ± 21.80 vs. 96.13 ± 23.37; p = 0.148), whereas CVB at the posterior pole was significantly higher in unaffected fellow eyes than normal control group (104.15 ± 23.35 vs. 103.31 ± 29.80; p = 0.040). CVD of entire fundus and posterior pole was higher in unaffected fellow eyes than normal control group (26.10 ± 2.34 vs. 24.68 ± 2.49; p = 0.029 and 25.97 ± 2.60 vs. 23.76 ± 1.97; p = 0.040) (Table 4).

**Table 4:** Comparison of choroidal parameters between unaffected fellow eyes of unilateral PCV and normal controls.

Variables	Unaffected fellow eye (n = 29 eyes)	Control (n = 27 eyes)	*p*-value
Subfoveal choroidal thickness (μm)	339.95 ± 64.78	276.42 ± 59.87	0.001
Haller’s layer	254.68 ± 50.92	180.65 ± 51.68	0.010
Choriocapillaris/Sattler’s layer	85.72 ± 22.74	94.42 ± 52.19	0.042
Choroidal vascular brightness intensity			
Total	101.69 ± 22.75	89.80 ± 22.54	0.012
Posterior pole	104.15 ± 23.35	103.31 ± 29.80	0.040
Peripheral region	93.98 ± 21.80	96.13 ± 23.37	0.148
Choroidal vascular density			
Total	26.10 ± 2.34	24.68 ± 2.49	0.029
Posterior pole	25.97 ± 2.60	23.76 ± 1.97	0.040
Peripheral region	28.98 ± 2.26	26.80 ± 2.83	0.058

*PCV* polypoidal choroidal vasculopathy.

### Choroidal vascular features including brightness intensity and associated factors

**Figure 2:**
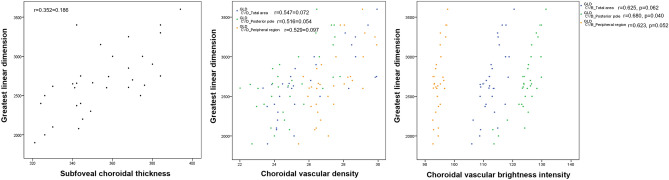
Scatter plot showing correlation between greatest linear dimension and choroidal vascular features.

In the Pearson correlation analysis, CVB at the posterior pole, peripheral region, and total area correlated significantly with SFCT and the number of polyps (all p < 0.05). Although CVB at the posterior pole correlated with the greatest linear dimension, there was no correlation between CVB at the peripheral region or the total CVB with the greatest linear dimension (p = 0.040, p = 0.052, and p = 0.067, respectively) CVD at the posterior pole, peripheral region, and total area correlated significantly with SFCT, especially Haller’s layer and the number of polyps (all p < 0.05). There was no correlation between CVD with age, choriocapillaris/Sattler’s layer, and the greatest linear dimension (Table 5). In addition, relationship of greatest linear dimension with choroidal vascular features was assessed using Person correlation (Fig. 2). A positive correlation was found between greatest linear dimension and SFCT or CVD in all regions which was not statistically significant. Greatest linear dimension was positively correlated with CVB at posterior pole, whereas no significant correlation was found with CVB at peripheral region and CVB of total area.

**Table 5:** Pearson correlation analysis of choroidal vascular features and associated factors.

	Choroidal vascular brightness intensity						Choroidal vascular density					
	Posterior pole		Peripheral region		Total		Posterior pole		Peripheral region		Total	
	R	*p*-value	R	*p*-value	R	*p*-value	R	*p*-value	R	*p*-value	R	*p*-value
Age	− 0.261	0.587	− 0.257	0.574	− 0.209	0.529	− 0.256	0.430	− 0.165	0.512	− 0.184	0.568
Subfoveal choroidal thickness	0.502	0.005	0.451	0.030	0.512	0.020	0.439	0.030	0.397	0.012	0.429	0.020
Haller’s layer	0.417	0.023	0.510	0.041	0.413	0.043	0.348	0.042	0.435	0.035	0.401	0.040
Choriocapillaris/Sattler’s layer	− 0.031	0.060	− 0.055	0.067	− 0.289	0.064	− 0.021	0.070	− 0.036	0.059	− 0.029	0.084
Central macular thickness	0.321	0.198	0.268	0.245	0.316	0.185	0.223	0.164	0.216	0.224	0.284	0.196
Polyp number at baseline	0.366	0.030	0.412	0.012	0.671	0.004	0.384	0.012	0.263	0.037	0.362	0.042
Greatest linear dimension	0.680	0.040	0.623	0.052	0.625	0.062	0.516	0.054	0.529	0.097	0.547	0.072

*CVB* choroidal vascular brightness, *CVD* choroidal vascular density, *SFCT* subfoveal choroidal thickness.

### Associations of phenotypes of PCV with the choroidal vascular features

In the univariate regression analysis, thickness of Haller’s layer, CVB in all regions, CVD of total area, and CVD at posterior pole were associated with the polyp number and the greatest linear dimension (all p < 0.05). In the multivariate analysis with linear regression analysis, CVB of total area and CVB at posterior pole were associated with polyp number (both p = 0.001). CVB of total area, CVB at posterior pole, and CVD at posterior pole were associated with greatest linear dimension (p = 0.013, p = 0.010, and p = 0.019, respectively) (Table 6).

**Table 6:** Univariate and multivariate linear regression analysis of factors related to phenotypes of PCV.

	Polyp number				Greatest linear dimension			
	Univariate		Multivariate		Univariate		Multivariate	
	β	*p*-value	β	*p*-value e	β	p value	β	*p*-value
Age	0.423	0.546			0.598	0.515		
Spherical equivalent	0.282	0.641			0.279	0.593		
Subfoveal choroidal thickness	0.347	0.147			0.340	0.127		
Haller’s layer	0.362	0.040	0.339	0.165	0.315	0.013	0.247	0.178
Choriocapillaris/Sattler’s layer	0.229	0.054			0.211	0.063		
Central macular thickness	0.231	0.499			0.167	0.450		
Choroidal vascular brightness intensity								
Total	0.540	0.010	0.496	0.001	0.478	0.030	0.447	0.013
Posterior pole	0.575	0.002	0.484	0.001	0.581	0.026	0.499	0.010
Peripheral region	0.466	0.013	0.312	0.055	0.504	0.020	0.475	0.053
Choroidal vascular density								
Total	0.349	0.026	0.318	0.147	0.351	0.042	0.247	0.135
Posterior pole	0.379	0.041	0.362	0.128	0.392	0.036	0.304	0.019
Peripheral region	0.326	0.053			0.223	0.061		

Multivariate model adjusting for factors significant in univariate analysis at p < 0.05.

*PCV* polypoidal choroidal vasculopathy.

## Discussion

In this study, CVB as seen on ICGA was investigated in terms of its association with different clinical phenotypes in patients with PCV. CVB of the entire fundus was higher in PCV than control eyes, predominantly in the posterior pole and inferior quadrants. In patients with unilateral PCV, CVB at the posterior pole was significantly higher in affected than unaffected fellow eyes, whereas CVB at the peripheral region did not differ between the two eyes. Also, CVB at the posterior pole correlated significantly with the SFCT, number of polyps, and greatest linear dimension. CVB at the posterior pole showed a better correlation with greatest linear dimension than SFCT or CVD.

In our patients, CVB was higher in PCV eyes than in the eyes of the age-matched control group, regardless of the segmented region. CVB was higher at the posterior pole than in the peripheral region, and higher in the inferior than superior quadrant in both the PCV and control group. Generally, nonuniform brightness intensity on ICGA is due to the amount of dye injected and an asymmetry in the rates of dye clearance. Matsumoto et al. demonstrated collateral vessel formation due to venous anastomosis in 27 of 30 eyes with treatment-naïve pachychoroid neovasculopathy (PNV), suggesting long-standing choroidal congestion^9^. In their patients, dilated choroidal vessels with hyperpermeability corresponding to areas of venous anastomosis were observed on ICGA. Later, the same group reported intervortex anastomosis in 90.2% of central serous chorioretinopathy, 95.1% of PNV, and 100% of PCV patients^10^. Spaide et al. also suggested that intervortex anastomosis is a response to choroidal congestion involved in the pathogenesis of pachychoroid disease^5^. Intervortex anastomosis and dilated choroidal vessels with choroidal vascular hyperpermeability are compensatory mechanisms for choroidal congestion and may explain the higher CVB at the posterior pole in PCV eyes, with gravity explaining the higher CVB at inferior regions^11^. Furthermore, tighter, more circuitous drainage from the inferior vortex veins to the superior ophthalmic vein might lead to a lower rate of dye clearance and eventually influence the CVB at inferior regions in both PCV and normal eyes. These imbalances of choroidal venous drainage may gradually cause an overload of the vortex vein system, manifesting as a higher CVB on ICGA.

Several studies reported retinal and choroidal changes in the fellow eyes of patients with unilateral PCV. In a previous study, we demonstrated binocular concordance of vortex vein engorgement in unilateral PCV, as seen on UWF-ICGA^6^. Another study found alterations in the retinal pigment epithelium (RPE) and pachyvessels in 84% and 60% of the fellow eyes of patients with unilateral PCV, respectively^12^. Similarly, the CVB of the peripheral region did not differ between affected and fellow eyes, although at the posterior pole it was higher in affected eyes. These observations are consistent with the development of venous remodeling and intervortex venous anastomoses at the posterior pole in response to an imbalance of choroidal venous drainage, with subsequent overload of the vortex vein system beyond the physiologic range in eyes with an underlying predisposition manifesting as disease. In fact, in an animal model, suturing only one vortex vein had little influence on choroidal structural changes. There might be more extensive disturbance of choroidal drainage system to a level overwhelming the normal drainage^13^. Interestingly, CVB and choroidal thickness at the posterior pole were greater in unaffected eyes than control eyes. The increased CVB in the peripheral region of PCV eyes might be related to choroidal outflow disturbance, which is a predisposing factor for unilateral PCV. A recent study proposed venous overload choroidopathy as the common pathophysiology in pachychoroid diseases^14^. While changes such as venous dilation, vascular remodeling, and anastomosis formation are insignificant if short-term or mild, secondary effects such as RPE damage can appear if the physiological range is exceeded or the changes are prolonged.

An analysis of the association between CVB and other clinical features showed that CVB correlated positively with SFCT, the number of polyps, and the greatest linear dimension. However, CVD was correlated with SFCT and the number of polyps, while it was not correlated with greatest linear dimension. These findings suggest that the degree of choroidal congestion is involved in the pathogenesis and phenotypes of PCV. Furthermore, the increased CVB representing dye retention may be an indicator of choroidal outflow congestion resulting from impaired venous drainage, with the subsequent inner choroidal attenuation producing an ischemic microenvironment that promotes vascular endothelial growth factor (VEGF) expression. In turn, these manifestations may lead to RPE alterations and polyp development. A correlation of SFCT with the PCV lesion area, and the influence of this relationship on the response of anti-VEGF treatment outcomes, have been reported^2,15^. A recent study using multimodal imaging demonstrated that total and polypoidal lesion areas tend to be larger in eyes with a higher SFCT^16^. However, the association of various choroidal features and PCV treatment outcomes is still unclear^17–20^.

Recent imaging studies have suggested that choroidal vascular hyperpermeability is followed by choriocapillaris atrophy and clinical manifestations such as RPE alteration and pigment epithelial detachment^8,21^. The area of choroidal vascular hyperpermeability usually overlaps with the region of the dilated vortex vein as seen on OCT images^22–25^. This area of choroidal hypercyanescence includes not only dilated choroidal vessels, but also dye leakage on conventional ICGA. In a quantitative analysis of brightness intensity using UWF-ICGA images and extracting only the choroidal vasculature, we avoided any influence on the results of potential confounding factors, such as axial length, refractive error, and differences in brightness signals caused by dye leakage.

Our study had several limitations. First, it used a retrospective design, and the sample size was relatively small. Second, middle-phase UWF ICGA images, in which the vortex veins are most visible, were selected for the analysis. Since only one eye can be imaged at a time, the timing of image acquisition might have differed between participants, and even between affected and unaffected fellow eyes. And there are possibilities that the polyps themselves may have affected the brightness. To overcome these limitations, the brightness of the retinal vessels around the disc was adjusted so that it matched on all of the images. Third, all participants were Korean; whether the results are generalizable to PCV in other ethnic groups remains to be determined. Fourth, because axial length was not collected in all patients, eyes with spherical equivalent greater than ± 6.00 diopters were excluded to minimize the effect of axial length. In this study, mean spherical equivalent was − 0.24 and it is likely that eyes with extremely short or long axial lengths were not included. Nevertheless, our results provide a detailed picture of the entire vortex vein system, as well as new insights into choroidal outflow congestion and its clinical significance.

In conclusion, we developed a method to analyze choroidal outflow by extracting the choroidal vessels and quantifying brightness intensity on UWF-ICGA images. Using this method, we observed a higher CVB in PCV than normal control eyes regardless of the region. In patients with unilateral PCV, affected eyes had a higher CVB at the posterior pole than unaffected fellow eyes, although there was no difference in the peripheral region. In addition, CVB correlated with SFCT, the number of polyps, and the greatest linear dimension. CVB at posterior pole showed positive correlation with greatest linear dimension, which was not correlated with SFCT or CVD. Determination of CVB in a manner that shows dye retention and clearance can serve as a new biomarker providing valuable information on clinical phenotypes of PCV. This objective and quantitative technique will also be useful for understanding other pachychoroid spectrum disease.

## Methods

### Study population

The medical records of patients with treatment-naïve PCV, which included subfoveal lesions diagnosed between January 2021 and November 2022, were retrospectively reviewed. This study was approved by the Internal Review Board of Yeungnam University Medical Center (IRB No: 2022-12-017) and adhered to the tenets of the Declaration of Helsinki. Informed consent was obtained from all subjects involved in the study. The diagnosis of PCV was made based on the presence of a polypoidal choroidal vascular lesion on OCT and ICGA image. The exclusion criteria were a history of other retinal diseases (including diabetic retinopathy, uveitis, retinal vascular occlusion, and pathologic myopia), spherical equivalent greater than ± 6.00 diopters, presence of contrast agent opacities or poor-quality images, massive subretinal hemorrhage or fibrosis obscuring the choroidal vasculature on ICGA, and a history of any vitreoretinal surgery or intravitreal injection. At baseline, all patients underwent a comprehensive ophthalmic examination, including best-corrected visual acuity (BCVA) measurement, dilated fundus examination, and spectral-domain OCT (Spectralis; Heidelberg Engineering, Heidelberg, Germany). Fluorescein angiography (FA) and ICGA were performed using an Optos California (Optos PLC, Dunfermline, UK) system. Fellow eyes in patients with unilateral epiretinal membrane who underwent FA and ICGA as part of a checkup that confirmed based on the history and ophthalmic examination were enrolled as age-matched control group.

### OCT image acquisition and analysis

OCT images with minimal scan rotation/tilting were analyzed by two trained retinal specialists (MS and AJ) blind to each other’s measurements and the patient information. SFCT and Haller’s layer thickness were measured manually using 1:1 pixel scale images with calipers in the proprietary software (Heidelberg Eye Explorer; Heidelberg Engineering, Heidelberg, Germany). SFCT was defined as the vertical distance from the choroidal–scleral interface to Bruch’s membrane at the foveal center. Haller’s layer was defined as the outer choroidal layer containing large choroidal vessels. Haller’s layer thickness was measured from the inner border of the choroidal–scleral junction to the innermost point of the selected large choroidal vessel at the foveal center. The remaining choroidal thickness was considered as the choriocapillaris/Sattler layer. Interobserver correlation coefficients for SFCT and Haller's layer thickness were 0.906 and 0.897, respectively. Central macular thickness (CMT) was automatically determined as mean thickness in the central 1 mm Early Treatment Diabetic Retinopathy Study grid.

### UWF-FA and UWF-ICGA image acquisition and analysis

UWF FA and UWF-ICGA were performed simultaneously in patients intravenously injected with a mixture of 5 mL of 10% sodium fluorescein and 2 mL of 25 mg of indocyanine green. Images were obtained during the early (2–3 min after injection), middle (5–10 min after injection), and late (10–15 min after injection) phases of the angiogram. The UWF- FA and UWF-ICGA images included one or more vortex veins per quadrant to minimize the influence of the change in the brightness of the fluorescence caused by the tilt of the eye. Images with minimal artifacts and good centration were reviewed and selected for further analysis by two trained retinal specialists (MS and AJ). The images were cropped to remove eyelid and eyelash artifacts. UWF images from the middle phase were used to evaluate CVB. Before the CVB was calculated, the relative brightness of the images was adjusted using the brightness of the retinal vessels in the optic nerve as a reference, to avoid potential confounding effects. Image segmentation was performed using ImageJ (NIH, USA, http://rsbweb.nih.gov/ij/) software. Otsu’s method is a variance-based binarization technique to find the threshold value that the weighted variance between the foreground and background pixels is the least according to its gray scale characteristics. Adaptive threshold method determines the threshold for a pixel based on a small region around it. This method provides different thresholds for different regions of the same image which gives better results for images with varying brightness intensity. The Otsu threshold method was applied for foreground extraction, and the adaptive threshold method was used for the extraction of high-intensity areas^26^.

**Figure 3:**
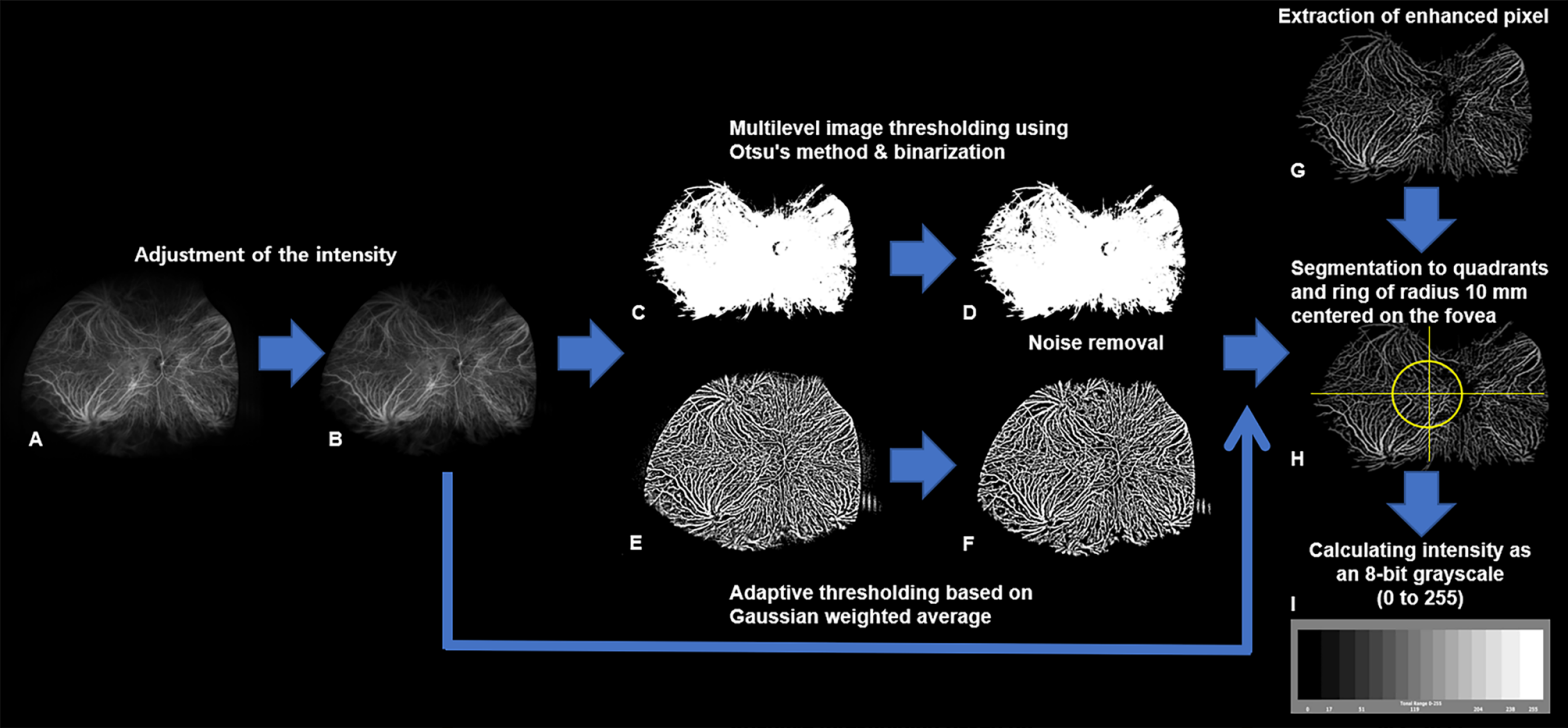
Image processing method mentioned in this study using ImageJ (NIH, USA, http://rsbweb.nih.gov/ij/) software. The original image was cropped to remove the background and areas obscured by the eyelashes (**A**). The brightness intensity of the entire image was adjusted so that the average intensity of the blood vessels inside the disc was the same in all images (**B**). Multilevel image thresholding, using Otsu's method and binarization, was employed to remove non-enhanced pixels (**C**,**D**). An adaptive threshold method based on the Gaussian-weighted average was applied to extract enhanced pixels (**E**,**F**). After noise removal (**D**), the image was enhanced (**F**,**G**). The images were segmented into quadrants within a circle with a radius of 10 mm centered on the fovea (**H**). The brightness intensity of each area was calculated as an 8-bit gray-scale (**I**).

Overlapping regions of these two images and the initial intensity-adjusted image were used to extract enhanced pixels of choroidal vessels. Retinal vessels extracted from the UWF-FA images were subtracted from the adjusted image. The images were segmented into quadrants and a circle with a radius of 10 mm drawn, centered on the fovea. The area inside the circle was defined as the posterior pole, and the areas outside the circle as the periphery. The brightness intensity in each area was averaged as an 8-bit grayscale (0–255) (Fig. 3).

Choroidal vascular density (CVD) was calculated using previously described methods^7^. Briefly, the early phase images were transformed into stereographic projection images using the manufacturer’s software (Optos PLC, Dunfermline, UK). UWF-FA and UWF-ICGA images were binarized after the choroidal vessels had been enhanced without noise using the top-hat filter embedded in imageJ software. The choroidal vascular area was calculated by subtracting the retinal vascular area of the FA images from the total vascular area of the ICGA images. All areas were measured by imageJ software. And all vascular areas were calculated in mm^2^ using the pixels. CVD was calculated by dividing the manually outlined choroidal vascular area by the total visible area. Inter-grader reliability was high for all annotations. For CVB and CVD, the intra-class correlation coefficients were 0.949 and 0.920, respectively.

### Sample size and statistical analysis

The number of subjects was calculated using G-power version 3.1 (Heinrich Heine University, Dusseldorf, Germany). The effect size of 0.8 was used and alpha was set at 0.05. The sample size of 27 in each group was required to achieve power of 0.8.

The data were analyzed using SPSS software (version 21.0; IBM Corp., Armonk, NY, USA). Differences between the PCV and control groups were examined using Mann–Whitney U-test and Fisher’s exact test. Comparisons between the affected eye and unaffected fellow eye were performed using the Wilcoxon signed rank test and Fisher’s exact test. A Pearson’s correlation test was used to determine the correlation between choroidal vascular features such as CVB or CVD, and other variables. Univariate and multivariate analysis were used to evaluate factors associated with phenotypes of PCV. In all analyses, two-tailed p values of < 0.05 were considered to indicate statistical significance.

## Data Availability

The datasets used and/or analysed during the current study available from the corresponding author on reasonable request.
